# Minimally Invasive (Epivastus) Approach for Total Knee Arthroplasty

**DOI:** 10.17691/stm2023.15.4.02

**Published:** 2023-07-28

**Authors:** S.E. Sokolovskii, A.A. Zykin, N.N. Rukina, E.E. Malyshev

**Affiliations:** PhD Student, Department of Traumatology, Orthopedics and Neurosurgery named after M.V. Kolokoltsev; Privolzhsky Research Medical University, 10/1 Minin and Pozharsky Square, Nizhny Novgorod, 603005, Russia; Head of Traumatology and Orthopedics Department No.2, University Clinic; Privolzhsky Research Medical University, 10/1 Minin and Pozharsky Square, Nizhny Novgorod, 603005, Russia; Senior Researcher, University Clinic; Privolzhsky Research Medical University, 10/1 Minin and Pozharsky Square, Nizhny Novgorod, 603005, Russia; Professor, Department of Traumatology, Orthopedics and Neurosurgery named after M.V. Kolokoltsev; Privolzhsky Research Medical University, 10/1 Minin and Pozharsky Square, Nizhny Novgorod, 603005, Russia

**Keywords:** gonarthrosis, total knee arthroplasty, minimally traumatic approach in endoprosthesis replacement, epivastus, medial mediapatellar approach

## Abstract

**The aim of the study** was to assess the efficiency of a developed minimally invasive (epivastus) approach in total knee arthroplasty (TKA) by comparing its early results with those of a standard medial mediapatellar approach (MMPA).

**Materials and Methods:**

A single-center, comparative randomized prospective study involved 127 patients, who underwent TKA using MMPA (n=62) and a modified minimally invasive epivastus approach (n=65) within the period from January to December, 2022. The study groups were comparable by gender, age, BMI, gonarthrosis stage, and knee joint functioning parameters.

**Results:**

The surgery duration in the epivastus group was significantly lower compared to MMPA group (p<0.001). However, the interpretation of tissue trauma markers assessment appeared rather ambiguous. There were no statistically significant differences in lactate dehydrogenase (p=0.253). C-reactive protein, myoglobin, creatinine showed a significant increase in MMPA group (p<0.001; p=0.002 and p=0.048, respectively), while aspartate aminotransferase, creatine phosphokinase and ESR, in contrast, increased in the epivastus group (p<0.001; p=0.024 and p=0.010, respectively). Pain syndrome determined by VAS 3 days after the surgery was significantly lower in the epivastus group (p=0.006). The extent of blood loss appeared to be much greater in MMPA group (p=0.006). The joint function indicators on day 3 after the surgery were found to be better in the patients after TKA using an epivastus approach (p<0.001). The postoperative assessment of the endoprosthetic spatial orientation showed the indicators characterizing the correct endoprosthetic implantation to be comparable in both groups (p≥0.06).

**Conclusion:**

The present study demonstrated the efficiency of the developed minimally invasive (epivastus) approach in TKA. However, it should be taken into consideration that surgeons should take a training course to be able to accomplish a high-quality approach.

An ambiguous interpretation of tissue trauma markers assessment of performing minimally traumatic approaches requires terminology correction. It is probably necessary to change the approach to the approach marking and use the terms specifying minimal invasiveness and the reduction of muscle injury rather than soft tissues in general.

## Introduction

Total knee artroplasty (TKA) is widely used in treating patients with terminal gonarthrosis and when conservative therapy fails [1-5]. The most common approach in TKA is medial mediapatellar approach (MMPA) described by Von Langenbeck as early as in 1874 [4]. The proportion of MMPA accounts for 91.9% from all TKA cases [6]. The approach has a number of advantages: it is universal, with sufficient imaging of the joint under surgery, and extendable. The main drawbacks of MMPA are the following: the injury of patellar tendon and neurovascular structures, which can result in anterointernal knee joint neuropathy and patellar circulation impairment [1, 7–9].

The best known minimally traumatic approaches, midvastus and subvastus, are used less frequently (4%) than MMPA [6]. According to literature data [1, 7–11], less injured extensor mechanism and muscle tone preservation observed in MMPA contribute to better TKA outcomes leading to hospital stay reduction, the postoperative pain intensity decrease, and quicker knee functioning recovery. However, with all their advantages, minimally invasive approaches are technically more complex, which has an impact on learning curve duration, insufficient surgical area visibility hinders correct positioning of prosthetic components. In addition, their usage in some cases is limited by patient’s anatomic features and entails the risk of neurovascular damage [8]. The mentioned disadvantages gave occasion to develop a novel approach for TKA and carry out the present study.

**The aim of the study** was to assess the efficiency of a developed minimally invasive (epivastus) approach in total knee arthroplasty by comparing its early results with those of a standard medial mediapatellar approach.

## Materials and Methods

### Study design

A single-center, comparative randomized prospective study included 127 patients. Within the period from January to December, 2022, the patients underwent TKA using MMPA (n=62) and a developed minimally traumatic epivastus approach

(n=65). The study was carried out in accordance with the declaration of Helsinki (2013) and approved by the Ethics Committee of Privolzhsky Research Medical University (Nizhny Novgorod, Russia). All patients gave their informed consent.

Inclusion criteria:

gonarthrosis, III degree according to Kosinskaya,s classification;

patients available for observation and control.

Exclusion criteria:

knee joint contracture (knee angle less 25°);

previous open knee surgeries;

fixed valgus deformity of the knee joint;

presence of systemic processes affecting connective tissue.

One of the key tools to assess knee joint functioning was the international modified scale, KOOS (Knee Injury and Osteoarthritis Outcome Score).

To study spatial and temporal characteristics of gait (2D gait analysis), kinematic indices (movement of the knee and ankle joints) in different walking phases (stance phase, swing phase, and a double step period), the registration of patient’s efforts and determination of optimal speed characteristics with a deviation relating to target values, Simi Motion Systems (SIMI, Germany) in 2D mode was used.

The groups under study were comparable by gender, age, BMI, gonarthrosis stage, joint functioning indices (Tables 1–3).

**Table 1:** Characteristic of the patients with total knee arthroplasty using the approaches under study, M±SD

Parameters	MMPA (n=62)		Epivastus (n=65)		p
Age (years)	63.56±8.12 (35–78)*		65.98±8.12 (43–84)*		0.095
BMI	34.0±5.90 (21.45–48.43)*		33.16±5.08 (21.82–44.73)*		0.390
Gender (abs. number/%)	Male	female	Male	female	0.91
	10/6.2	52/93.8	11/7.15	54/92.85	

* maximal and minimal values are given for the parameters “Age” and “BMI”.

**Table 2:** Preoperative characteristics of knee joint functioning (according to KOOS), Me [Q1; Q3]

Scale parameters	MMPA (n=62)	Epivastus (n=65)	p
Pain	44.0 [32.0; 54.0]	44.4 [33.3; 55.5]	0.48
Symptoms	51.2 [41.0; 57.1]	46.4 [35.7; 57.1]	0.15
Everyday physical activity	48.4 [37.0; 58.0]	44.1 [35.3; 69.1]	0.80
Physical activity in doing sport	35.0 [0; 60.0]	20.0 [15.0; 42.5]	0.34
Life quality	23.9 [6.3; 31.2]	15.6 [0; 31.3]	0.55
Total score	38.8 [33.0; 45.4]	33.8 [28.4; 50.6]	0.20

**Table 3:** Preoperative knee joint functioning in the studied groups by gait analysis (2D gait analysis), Me [Q1; Q3]

Gait parameters	MMPA (n=62)	Epivastus (n=65)	p
Walking pace (km/h)	3.6 [3.3; 4.0]	3.5 [3.0; 4.6]	0.83
Stance phase (%)	69.6 [67.6; 73.1]	69.1 [66.2; 71.4]	0.08
Swing phase (%)	30.4 [26.9; 32.4]	31.0 [28.6; 33.8]	0.08
Deviation (%)	–9.6 [–13.1; –7.6]	–9.0 [–11.2; –6.2]	0.07
Double step (s)	1.3 [1.1; 1.6]	1.4 [1.2; 1.7]	0.14
Deviation (s)	–0.1 [–0.3; –0.2]	–0.03 [–0.2; 0.3]	0.16
Movements in the knee (degrees)	44.8 [32.2; 53.5]	42.6 [28.5; 52.8]	0.59
Deviation (degrees)	–25.7 [–36.2; 16.5]	–27.4 [–41.5; 17.6]	0.42
Movements in the ankle (degrees)	25.7 [21.2; 27.5]	25.6 [22,3; 29.3]	0.39
Deviation (degrees)	–4.3 [–8.7; –2.5]	–4.4 [–7.7; –0.7]	0.39

All surgical procedures were performed by one surgical team under the charge of an experienced surgeon who uses these approaches in routine practice.

### Surgery technique

The authors developed and tested a novel minimally traumatic approach (epivastus) in TKA (application for a patent No.2022126380). The approach was performed as follows (see the Figure). Skin incision was made above the kneecap and extended up to the tibial tuberosity; then a distal part of the medial vastus muscle was separated. Arthrotomy was performed from the proximal and middle third of the medial border of the patella (*2*) along its medial border leaving 5 mm inward, and along the patellar tendon up to the tibial tuberosity (*3*). Starting arthrotomy (*2*) the medial vastus muscle tendon tissues were incised passing to its muscular part and then going along the muscular fibers proximally and posteriorly so that to leave no more than 1 cm (*1*) from the distal muscular part of quadriceps head.

**Figure:**
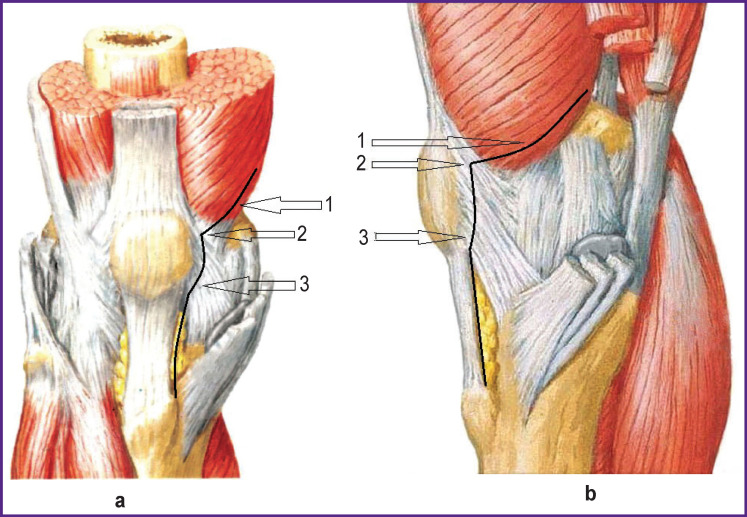
The approach scheme, frontal (*a*) and lateral (*b*) views For an explanation, see the text

A MMPA was performed according to a standard technique.

After performing both approaches, the soft tissue release was made; using special tool kit there was performed a modeling resection of the kneecap, the femoral and tibial condyles; symmetrical extension and flexion intervals being formed; original prosthetic components were installed followed by layered closure of the operative wound tightly with 24-h drainage.

As a prototype for a novel approach, we chose midvastus approach, when arthrotomy is performed from the upper inner angle of the patellar, then medially along the patellar ligament up to the tibial tuberosity. The vastus medialis is incised along the muscular fibers at an angle of 45–50° for 4.5–6.0 cm.

### Estimated results

The surgery traumatism was analyzed by the overall tissue damage in total knee arthroplasty using the following tissue trauma markers: C-reactive protein (CRP), creatine phosphokinase (CPK), lactic dehydrogenase (LDH), aspartate aminotransferase (AST), creatinine, myoglobin, and erythrocyte sedimentation rate (ESR). These markers of tissue trauma were assessed preoperatively and on day 3 after the surgery. Within the time period specified, we performed full blood count to determine the objective volume of blood loss (*V*_bl_) after the surgery. Blood loss volume was estimated by a modified Moore formula [6]:

Vbl(ml)=CBVref(Htref−Htact)/Htref,

where CBV_ref_ — reference circulating blood volume (females — 60 ml/kg, males — 70 ml/kg, obese — 75 ml/kg); Ht_ref_ — reference hematocrit (males — 45% (0.45), females — 42% (0.42)); Ht_act_ — actual hematocrit.

Pain syndrome was assessed using Visual Analogue Scale (VAS) on day 3 after the operation.

Early postoperative knee functioning was estimated using knee joint range of motions (neutral zero method) and, wherever possible, active elevation of a straight leg in supine position.

The prosthetic components positioning was assessed by the following reference angles: aLDFA — anatomical lateral distant femoral angle, MPTA — medial proximal tibial angle, TFA — tibiofemoral angle, as well as by the tibial and femoral pitches in the sagittal plane.

The data were ***statistically processed*** using Statistica 10.0 and Microsoft Office Excel. The normalcy of distribution was determined by Kolmogorov–Smirnov test. The study results were represented as Me [Q1; Q3] for quantitative indices, and in percentage — for qualitative values. In some cases, for illustrative purposes the indices were represented as M±SD, where M is mean characteristic value, SD is standard deviation; for some data, we demonstrated minimal and maximal values. To assess the findings, we applied paired t-test for dependent and independent groups, as well as Mann–Whitney U test. Fisher–Yates test was used for frequency analysis of contingency tables. The differences were considered statistically significant if p<0.05.

## Results

The surgery duration in the epivastus group was 45.0 [40.0; 50.0] min, which was significantly lower compared to that in the patients after TKA using MMPA — 55.0 [50.0; 63.0] min (p<0.001). Pain syndrome on day 3 after the operation and assessed by VAS was significantly lower in the epivastus group than in MMPA group, and accounted for 2.0 [1.0; 2.0] and 3.0 [2.0; 4.0], respectively (p=0.006). The blood loss analysis revealed a significant difference between two patients groups: in MMPA group, blood loss volume was 1225.79 [880.0; 1671.10] ml, and was significantly higher compared to that in the epivastus group: 986.01 [676.25; 1312.20] ml (p=0.006).

The values of all tissue trauma markers 24 h prior to the surgery were comparable (p>0.05). However, the analysis of the postoperative data showed ambiguous findings. Postoperative LDH showed no variation, while such markers as CRP, myoglobin, creatinine were significantly higher compared to those in MMPA group, whereas other markers such as AST, CPK, ESR, on the contrary, significantly increased in the epivastus group postoperatively (Table 4).

**Table 4:** Values of tissue trauma markers in the studied groups, Me [Q1; Q3]

Tissue markers trauma	MMPA	Epivastus	p
CRP (mg/L)	81.4 [76.1; 140.0]	70.7 [55.3; 76.7]	<0.001
CPK (U/L)	322.0 [181.0; 732.0]	575.5 [324.0; 895.0]	0.010
LDH (U/L)	357.5 [310.0; 421.0]	303.0 [142.0; 431.0]	0.253
AST (U/L)	30.4 [21.6; 37.0]	32.6 [27.5; 49.8]	0.024
Creatinine (μmol/L)	90.0 [80.0; 109.0]	82.8 [78.0; 91.1]	0.048
Myoglobin (μg/L)	76.8 [38.4; 154.0]	38.4 [28.8; 76.8]	<0.001
ESR (mm/h)	29.0 [23.0; 40.0]	58.0 [37.0; 65.0]	<0.001

The indices characterizing postoperative knee functioning in the patients with the epivastus approach of TKA (Table 5) were significantly better compared to those in MMPA patients (p<0.001).

**Table 5:** Muscle strength characteristics (the ability to lift straight leg in the supine position) and joint range of motions after total knee arthroplasty in the studied groups

Approach	Straight leg elevation (abs. number/%)			Flexion degree (degrees)	
	Yes	No	p	M±SD	p
MMPA (n=62)	39/61.90	24/38.10	0.0001	60.95±20.94	0.001
Epivastus (n=65)	63/96.92	2/3.69		85.92±23.25	

The spatial orientation analysis revealed no differences in both groups in prosthetic spatial orientation; the developed approach had no effect on the correctness of the installed prosthesis (Table 6).

**Table 6:** Positioning of prosthetic components in the approaches under study, Me [Q1; Q3]

Reference (degrees) angles	MMPA (n=62)	Epivastus (n=65)	p
aLDFA	84 [83; 85]	84 [83; 85]	0.7
MPTA	88 [88; 90]	89 [88; 90]	0.06
TFA	175 [174; 177]	175 [173; 177]	0.12
Tibial component angle	86 [84; 88]	85 [83; 87]	1.0
Femoral component angle	3 [2; 5]	4 [2; 5]	0.06

## Discussion

Most orthopedic surgeons use a standard mediapatellar approach in TKA [6]. It combines all the necessary requirements: sufficient visualization of the surgical area, it is universal, extendable, and easy-to-use [7-9]. Currently, the number of cases using minimally invasive approaches is few [6]. In addition, these approaches damage extensor mechanism far less, and so contribute to earlier knee joint functioning recovery.

Currently, there have been published many studies demonstrating the difference in patients’ functioning recovery, and in the changes in tissue trauma markers in TKA using a particular approach [7-15]. So, Niki et al. [15] compared the muscular damage in TKA using four approaches (3 minimally traumatic: midvastus, subvastus, quadriceps-sparing, and MMPA) and found a significant increase postoperatively in myoglobin and CPK in the midvastus group on days 7 and 9, respectively. Huang et al. [16] in their study analyzed soft tissue damage in total knee arthroplasty using a midvastus approach (n=30) and MMPA (n=30). They assessed such indices as CPK, myoglobin, LDH, glutamic oxalacetic transaminase, CRP, IL-6, and IL-1β before and after surgery. The midvastus group showed significant increase in CPK level in blood serum on days 2 and 3 after the operation. Moreover, MMPA patients demonstrated elevated CRP and IL-6 levels. Sabatini et al. [10] studied subvastus and MMPA groups and revealed a comparable increase of CPK and CRP (p=0.87) in both groups. Mean values of intraoperative blood loss were 1.9 L in a MMPA group and 1.45 L in a subvastus group, showing no significant differences (p=0.47). Our findings are comparable with those described in the above-mentioned studies. However, it is our opinion that minimal traumatism in the present study concerns the muscles exclusively rather than soft tissues in general as evidenced by a significant increase in myoglobin level in the MMPA group.

In its turn, the use of minimally invasive approaches results in better functional outcomes. Pan et al. [17] in their prospective randomized study compared the clinical consequences of TKA using a subvastus approach (n=35) and MMPA (n=33). The researchers noted less blood loss, higher functional outcomes in a group with a minimally invasive approach. Pain syndrome decreased as early as on the second postoperative day.

The analysis of the early outcomes of TKA using the epivastus approach showed a functional advantage over the standard technique as well.

However, it should be mentioned that the use of minimally traumatic approaches, with all their advantages, can influence the correctness of a prosthesis installed due to the limited visualization of an operable joint, and potentially can worsen a surgical result. An important factor contributing to the outcome is surgeon’s experience in performing TKA. The skills and abilities have a direct effect on surgery efficiency and complications, a learning curve of an operating surgeon plays a major role. Migliorini et al. [8] have similar views and confirmed in their study that minimally traumatic approaches are complex to accomplish and require a long training.

## Conclusion

The approach we developed for the primary total knee arthroplasty contributes to less traumatism of the extensor mechanism of a knee joint compared to the standard technique; it enables to preserve the tone and power of quadriceps muscle of thigh providing better functional outcomes in an early postoperative period.

To obtain positive results of using various approaches, it is necessary to assess objectively their technical application. It should be appreciated that minimally traumatic approaches present technical challenges, so it can affect both soft tissue damage and the correctness of a prosthesis installed; eventually, it can lead to poor functionally outcomes. An ambiguous interpretation of traumatism assessment of minimally invasive approaches requires terminology correction. It is probably necessary to change the way to the approach marking and use the terms specifying minimal invasiveness and the reduction of muscle injury rather than soft tissues in general.
